# ZD6474, a new treatment strategy for human osteosarcoma, and its potential synergistic effect with celecoxib

**DOI:** 10.18632/oncotarget.4179

**Published:** 2015-05-19

**Authors:** Jiani Liu, Jiangxue Wu, Ling Zhou, Changchuan Pan, Yi Zhou, Wuying Du, Jie-min Chen, Xiaofeng Zhu, Jingnan Shen, Shuai Chen, Ran-yi Liu, Wenlin Huang

**Affiliations:** ^1^ Sun Yat-Sen University Cancer Center, State Key Laboratory of Oncology in South China, Collaborative Innovation Center of Cancer Medicine, Guangzhou, China; ^2^ Department of Oncology, Jingzhou Hospital, Tongji Medical College of Huazhong University of Science and Technology, Jingzhou, Hubei, China; ^3^ Medical Oncology, Sichuan Cancer Hospital and Institute, Second People's Hospital of Sichuan Province, Chengdu, China; ^4^ Musculoskeletal Oncology Department, First Affiliated Hospital of Sun Yat-Sen University, Guangzhou, China; ^5^ Guangdong Provincial Key Laboratory of Tumor Targeted Drugs and Guangzhou Enterprise Key Laboratory of Gene Medicine, Guangzhou Doublle Bioproducts Co. Ltd., Guangzhou, China

**Keywords:** ZD6474, celecoxib, osteosarcoma, EGFR, cyclooxygenase-2

## Abstract

ZD6474, a small molecule VEGFR and EGFR tyrosine kinase inhibitor, has been considered as a promising tumor-targeted drug in various malignancies. EGFR and cyclooxygenase-2 (COX-2) were found overexpressed in osteosarcoma in previous reports, so here we tried to explore the anti-osteosarcoma effect of ZD6474 alone or combination with celecoxib, a COX-2 inhibitor. The data demonstrated that ZD6474 inhibited the growth of osteosarcoma cells, and promoted G1-phase cell cycle arrest and apoptosis by inhibiting the activity of EGFR tyrosine kinase, and consequently suppressing its downstream PI3k/Akt and MAPK/ERK pathway. Additionally, daily administration of ZD6474 produced a dose-dependent inhibition of tumor growth in nude mice. Celecoxib also significantly inhibited the growth of osteosarcoma cells in dose-dependent manner, while combination of ZD6474 and celecoxib displayed a synergistic or additive antitumor effect on osteosarcoma *in vitro* and *in vivo*. The possible molecular mechanisms to address the synergism are likely that ZD6474 induces the down-regulation of COX-2 expression through inhibiting ERK phosphorylation, while celecoxib promotes ZD6474-directed inhibition of ERK phosphorylation. In conclusion, ZD6474 exerts direct anti-proliferative effects on osteosarcoma cells, and the synergistic antitumor effect of the combination of ZD6474 with celecoxib may indicate a new strategy of the combinative treatment of human osteosarcoma.

## INTRODUCTION

Osteosarcoma is the most common primary malignant bone tumor in children and young adults [1, 2], which has been found to harbor a highly unstable genome and several important genomic alteration including multiple signaling pathways and microRNAs [2-5]. Although aggressive surgery, intensive radiotherapy and chemotherapy regimens (with agents such as doxorubicin, methotrexate, cisplatin, etoposide, and ifosfamide) have been utilized, the clinical outcome for osteosarcoma remains discouraging [6-8]. Moreover, traditional chemotherapy always leads to the enrichment or induction of osteosarcoma stem cells [9] and consenquently resulted in treatment resistance. Thus, novel therapeutic approaches targeting important pathways are urgently needed to improve the treatment of osteosarcoma [7, 10, 11].

Various overexpressed cell surface transmembrane receptors, such as ErbB2, EGFR, IGF-1R, PDGFR and VEGFR, activate diverse signaling cascades that have been implicated in osteosarcoma oncogenesis [12-15]. Certain targeted small molecule inhibitors have been expected to lead an efficient treatment in osteosarcoma [16]. However, gefitinib and BIBW2992, two EGFR inhibitors, were reported not effective against osteosarcoma cells in a recent report, though osteosarcoma cells do over-express EGFR [14]. So further studies are necessary to explore the potential of other therapeutic agents targeting EGFR [14]. ZD6474 is a small molecule inhibitor targeting multiple tyrosine kinases, including VEGFR-2 and EGFR) [17], which can block multiple intracellular signaling pathways involved in tumor growth, progression, and angiogenesis in several tumors [17-19]. In this study, we firstly tried to explore whether ZD6474 demonstrated a potential therapeutic efficacy in the treatment of osteosarcoma.

COX-2 was also found overexpressed frequently in osteosarcoma, which promoted malignant potential of neoplasms [20, 21] and was associated with poor prognosis for patients with osteosarcoma [22, 23]. Celecoxib, a selective COX-2 inhibitor, can induce osteosarcoma cell apoptosis via inhibiting COX-2 [24, 25], and has been applied for the clinical trials to treat osteosarcoma and Ewing sarcoma [26, 27]. Here, we also investigate the anti-proliferative effect of combined treatment with celecoxib and ZD6474 on human osteosarcoma *in vitro* and the antitumor activity on osteosarcoma xenografts *in vivo*, to search a potential efficient comprehensive therapeutics for osteosarcoma.

## RESULTS

### ZD6474 inhibits the proliferation of human osteosarcoma cells

**Figure 1:**
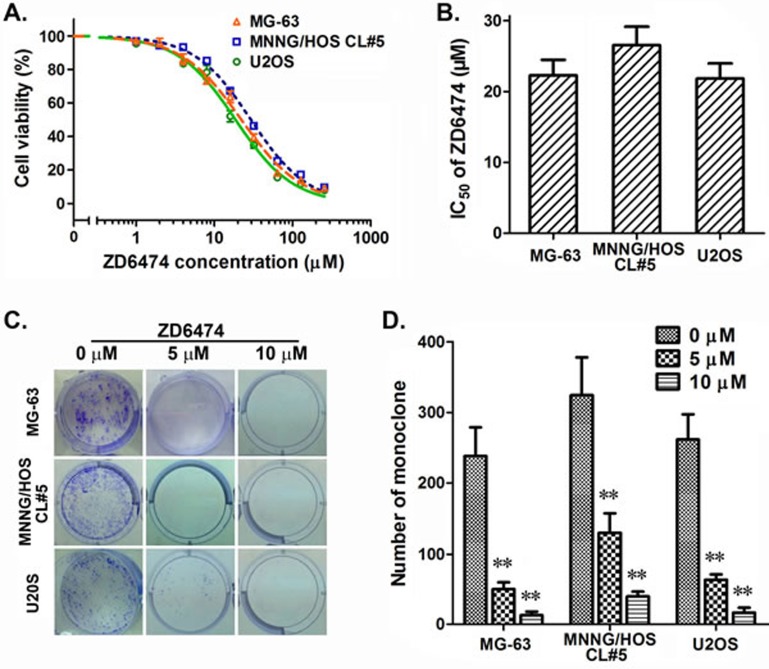
Antiproliferative effects of ZD6474 on human osteosarcoma cell lines **A. B.** The MTT assay. **A**, Dose-dependent curves (one of representative data). Data are given as relative cell viabilities compared with untreated control group. **B**, IC50 values of ZD6474. Data are shown as averages from 3-4 independent experiments. **C.**, **D.** The clone formation assay. **C**, representative pictures; **D**, The average of monoclone number. Data shown are from 3-4 independent experiments. **p < 0.01 compared with control groups (0 μM) (one-way ANOVA).

The anti-proliferation effect of ZD6474 on osteosarcoma cells was measured by MTT assay and clone formation assay. The data demonstrated that ZD6474 efficiently inhibited the proliferation of osteosarcoma cells (Figure 1A), and the half maximal inhibitory concentrations (IC50) of ZD6474 in MG-63, MNNG/HOS CL#5, and U2OS cells were 22.26±3.82, 26.55±5.20 and 21.85±3.65 μM, respectively, after treated for 72 hours (Figure 1B). Meanwhile, the clone formation of 3 osteosarcoma cells was all significantly inhibited by ZD6474 (Figure 1C and 1D) (*p* < 0.01). In summary, ZD6474 inhibited the proliferation of osteosarcoma cells in a dose-dependent manner.

### ZD6474 induces cell cycle arrest in human osteosarcoma cells

**Figure 2:**
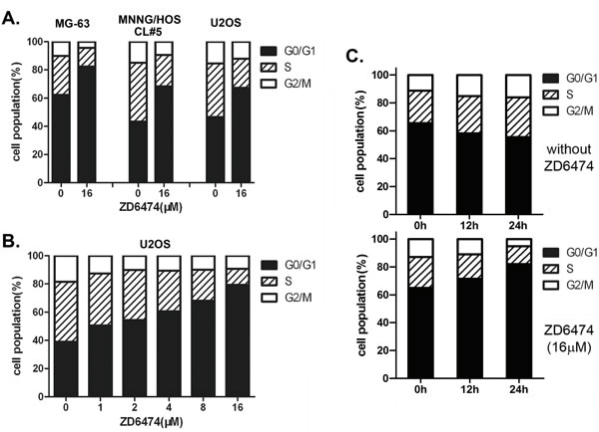
ZD6474 induces cell cycle arrest in human osteosarcoma cell lines **A.** FACS analysis of the cell cycle distribution of osteosarcoma cells after treatment with ZD6474 for 24 hours. ZD6474 delays G1-S cell cycle progression in osteosarcoma cells. **B.** FACS analysis of the cell cycle distribution of U2OS cells after treated with increasing concentrations of ZD6474. **C.** FACS analysis of the cell cycle distribution of synchronized MG-63 cells treated with 16 μM ZD6474 (bottom) or not (top) at 0, 12, 24 hours.

To examine whether the antiproliferative effect of ZD6474 on osteosarcoma cell lines was mediated via specific cell cycle arrest, we investigated the cell cycle phase distribution by flow cytometric analysis after ZD6474 treatment. We found that there was an obvious accumulation of cells during G0/G1 phase in MG-63, MNNG/HOS CL#5, and U2OS cells treated with 16 μM of ZD6474 for 24 hours (Figure 2A). Next, we incubated U2OS cells with ZD6474 at gradient concentrations, and found that the percentages of cells during G0/G1 phase were increased with increasing concentration of ZD6474 within a certain range (Figure 2B). Moreover, the G1-phase cell cycle arrest induced by ZD6474 was also time-dependent in U2OS cells (data not shown).

To further dissect the G1-S cell cycle arrest induced by ZD6474, MG-63 cells were re-entered into the cell cycle by addition of serum, and cell cycle progression was monitored in the presence or absence of ZD6474 after synchronized in G0/G1 phase by serum starvation overnight. The results showed that untreated cells rapidly exited G0/G1 phase and went through S phase, oppositely ZD6474-treated cells showed an accumulating during G0/G1 phase (Figure 2C). That is to say, cells during G0/G1 phase gradually increased in ZD6474-treated group while decreased in control group after serum recovery (12 hours, 71.44% verse 58.14%; 24 hours, 82.09% vserse 55.45%). These data suggested that ZD6474 induced G1/S phase cell cycle arrest in human osteosarcoma cells.

### ZD6474 promotes apoptosis in human osteosarcoma cells

**Figure 3:**
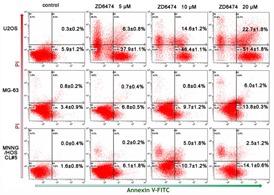
Apoptosis analysis by Annexin-V/PI double staining Annexin-V/PI double staining was performed on three osteosarcoma cells after treated with ZD6474 at 0, 5, 10 or 20 μM for 48 hours. The graphs are representative of two duplicate experiments, and the percentages of Annexin-V-positive (lower-right quadrant) and Annexin-V/PI double-positive (upper-right quadrant) from these experiments are shown in the relevant quadrants.

Osteosarcoma cells were treated with 0, 5, 10, or 20 μM of ZD6474 for 48 hours, the annexin-V/PI binding assay was performed to investigate the influence of ZD6474 on cell apoptosis. The data showed that the percentage of early apoptotic cells (Annexin-V-positive) increased in osteosarcoma cells treated with ZD6474 compared with the control group (*p* < 0.05) (Figure 3). The percentages of early apoptotic cells in U2OS cells treated with 5 μM, 10 μM or 20 μM of ZD6474 were 37.9±1.1%, 46.4±1.1% and 51.4±1.8%, respectively, while that of the control group was only 5.9 ± 1.2% (Figure 3). Similar results were observed in MG-63 and MNNG/HOS CL#5 cells (*p* < 0.05). These data suggested that ZD6474 promoted apoptosis in human osteosarcoma cells in a dose-dependent manner.

### ZD6474 inhibits the activation of EGFR pathway

**Figure 4:**
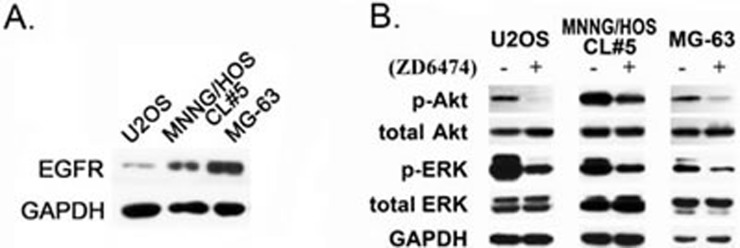
The effect on the EGFR downstream signaling pathways in response to ZD6474 **A.** EGFR expression in osteosarcoma cells. **B.** ZD6474 inhibits the phosphorylation of Akt and ERK in osteosarcoma cells. Cells were treated with 10 μM ZD6474 for 2 hours, and protein levels were checked by Western blotting analysis as described in Materials and Methods.

To explore the mechanisms by which ZD6474 inhibits the proliferation of osteosarcoma cells, EGFR expression was firstly evaluated at the protein level, and visible bands of EGFR protein was detected in all of osteosarcoma cells, and these is a high EGFR expression in MG-63 and MNNG/HOS CL#5 cells compared with that in U2OS cells (Figure 4A). After ZD6474 treatment for 2 hours, the phosphorylation of Akt and ERK decreased significantly in U2OS, MG-63 and MNNG/HOS CL#5 cells though total Akt and ERK proteins had no obvious changes (Figure 4B). These findings suggested that ZD6474 inhibited the phosphorylation of key signaling molecules (such as Akt, ERK) by blocking EGFR tyrokinase activity, and restrained the activation of two major downstream signal pathways, PI3K/Akt and MAPK/ERK.

### Combination treatment with ZD6474 and celecoxib inhibits the proliferation of osteosarcoma cells

**Figure 5:**
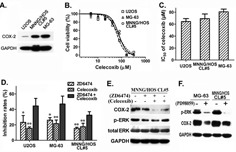
Combination treatment of ZD6474 and celecoxib inhibited the proliferation of osteosarcoma cells **A.** COX-2 expression in osteosarcoma cells. **B.**, **C.** MTT assay showed that celecoxib inhibited the proliferation of osteosarcoma cells in a dose-dependent manner. **B.** Dose-dependent curves (one of representative data). Data are given as relative cell viabilities compared with untreated control group. **C.** IC50 values of celecoxib. Data shown are from 3 independent experiments. **D.** MTT assay showed that the combination of ZD6474 and celecoxib exhibited stronger antiproliferative effect in osteosarcoma cells. *p < 0.05, **p < 0.01 compared with combination group (one-way ANOVA). **E.**, **F.** Western blotting analysis. Cells were treated with **E**. ZD6474 (2 hours) or/and celecoxib (24 hours) ERK inhibitor PD98059 (24 hours) protein levels were detected.

We measured COX-2 expression at the protein level in osteosarcoma cells and high level of COX-2 protein was found in MG-63 and MNNG/HOS CL#5 cells while lower expression in U2OS (Figure 5A). And COX-2 expression could be inhibited by celecoxib treatment within 24 hours in MNNG/HOS CL#5 cells (Figure 5E). MTT assay revealed that the proliferation of osteosarcoma MG-63, MNNG/HOS CL#5 and U2OS cells was inhibited by celecoxib treatment in a dose-dependent manner (Figure 5B), and IC50 values of celecoxib for 72 hours of incubation were 80.5±7.9, 69.1±13.5 and 64.3±8.6 μM, respectively (Figure 5C).

Considering that multi-target therapy always show stronger antitumor effect than single-target one, we examined the antiproliferation activity of ZD6474 combined with celecoxib, and found that combination treatment with ZD6474 (10 μM) and celecoxib (40 μM) displayed a significantly higher activity than treatment with ZD6474 or celecoxib alone (*p* < 0.05 or 0.01) (Figure 5D), and combined treatment showed additive effects in MG-63 and MNNG/HOS CL#5 cells (Q = 1.120 and 1.076) or synergistic effect in U2OS cells (Q = 1.268).

To clarify the possible mechanisms to address the synergistic or additive effect between ZD6474 and celecoxib, we checked COX-2, p-ERK levels after treated cells with ZD6474 or/and celecoxib. The results showed that not only celecoxib but ZD6474 down-regulated COX-2 expression, meanwhile celecoxib promoted the inhibition of ERK phosphorylation directed by ZD6474 though celecoxib alone had no obvious effect (Figure 5E). To make clear how ZD6474 caused COX-2 down-regulation, we checked the effect of ERK inhibitor (PD98059) on COX-2 expression, and found that the protein level of COX-2 was down-regulated in MNNG/HOS CL#5 cells and MG-63 cells after PD98059 treatment (Figure 5F). So we assume that ZD6474 and celecoxib likely produce an additive or synergistic anti-tumor effect on osteosarcoma via ZD6474-mediated the downregulation of COX-2 expression and celecoxib-directed the enhancement of ERK phosphorylation inhibition. Meanwhile, the inhibitory effect of ZD6474 on COX-2 expression is likely functioned via inhibiting MAPK/ERK pathway.

### Treatment with ZD6474 or/and celecoxib inhibits the growth of osteosarcoma xenografts in nude mice

**Figure 6:**
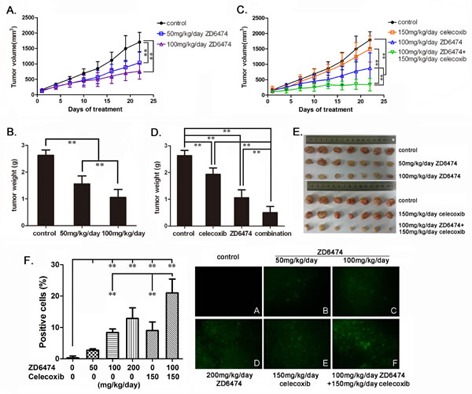
Antitumor effect of ZD6474 alone or in combination with celecoxib on human osteosarcoma xenografts in BALB/c nude mice MNNG/HOS CL#5 xenografts were established, treated, and monitored as described in Materials and Methods (*n* = 7/group). **A.** The growth curves of osteosarcoma xenografts under ZD6474 treatment. Data points represent an average from 7 mice (Bars, SD). A significant dose-dependent reduction of tumor volume was observed (**p < 0.01, two-way ANOVA). **B.** Tumor weights of xenografts after ZD6474 treatment (**p < 0.01, one-way ANOVA). **C.** The growth curves of osteosarcoma xenografts under ZD6474 or/and celecoxib treatment. Growth of tumors was significant slower in the ZD6474 and celecoxib combination treatment group than those in the control, ZD6474 or celecoxib single treatment groups (**p < 0.01, two-way ANOVA). **D.** Tumor weight of xenografts after ZD6474 or/and celecoxib treatment (**p < 0.01, one-way ANOVA). **E.** Picture of resected xenografts. **F.** TUNEL assays. The percentages of apoptotic cells (with green fluorescence) were presented as average ± SD (Left) (**p < 0.01, one-way ANOVA), and the representative pictures (×200) were displayed (Right).

Consequently, we investigated antitumor effect of treatment with ZD6474 and/or celecoxib in nude mice suffering MNNG/HOS CL#5 osteosarcoma xenografts. The results showed that the growth of tumors in the groups treated with ZD6474 was significantly slower than that in the control group (*p* < 0.01) (Figure 6A). Once-daily oral administration of ZD6474 produced a significant dose-dependent inhibition effect. Inhibition rates were 59.5% and 39.8% in the groups treated with ZD6474 100 or 50 mg/kg/day respectively (*p* < 0.01) (Figure 6B). Celecoxib (150mg/kg/day) alone also significantly inhibited the growth of osteosarcoma xenografts (*p* < 0.01) (Figure 6C, 6D). Moreover, administration of ZD6474 (100 mg/kg/day) combined with celecoxib (150mg/kg/day) displayed more antitumor effects compared with ZD6474 or celecoxib alone (*p* < 0.01) (Figure 6C). The inhibition rates were 81.1% (combination group) vs. 59.5% (ZD6474 alone) or 26.3% (celecoxib alone), respectively (*p* < 0.01) (Figure 6D). The combined treatment showed a synergistic antitumor effect (Q = 1.156). There were not obvious effects on the body weight of mice in animal studies described above (data not shown), indicating that single-agent ZD6474 and ZD6474 combined with celecoxib are likely well-tolerated.

After treated for 22 days, xenografts were resected (Figure 6E) and cell apoptosis was analyzed by TUNEL assay. There were obvious cell apoptosis (cells with green fluorescence indicating apoptotic cells) found in all tumor sections from groups treated with ZD6474, celecoxib alone or combination, while few apoptotic cells in the control group(Figure 6F). The percentages of apoptotic cells increased in ZD6474-treated groups with ZD6474 dose increasing, and that in the group treated with the combination of ZD6474 and celecoxib was significantly higher than those in the groups treated with ZD6474 or celecoxib alone (Figure 6F) (*p* < 0.01).

## DISCUSSION

Growth-inducing signaling is needed for cells to move from a quiescent state into an active proliferative state. Growth factor receptors overexpressed in many cancers carry tyrosine kinase activity in their cytoplasmic domains and transduce stimulatory growth signals into the cell interior. Inhibition of tyrosine kinases blocks multiple intracellular signaling pathways involved in tumor growth, progression, and angiogenesis [28]. Osteosarcoma expresses cell surface transmembrane receptors that share common features of transmembrane receptors with tyrosine kinase activity, and these receptors activate different intracellular signaling cascades that have been implicated in oncogenesis [29, 30]. ZD6474 is a novel VEGFR-2, EGFR and Src tyrosine kinase inhibitor, which suppresses a number of tumors, including non-small cell lung cancer as well as breast, gastric, prostate, colorectal, nasopharyngeal cancers and leukemia, through inhibiting tumor cell growth and survival [31-34].

The expression of EGFR was detected in the vast majority of osteosarcoma [14]. We also found that EGFR was expressed in all of three osteosarcoma cell lines used in this study, though the EGFR protein level in U2OS cells was markedly lower than in other two cell lines. Although gefitinib and BIBW2992, two EGFR inhibitors, were reported not effective against osteosarcoma cells [14], we found in this study that ZD6474 exerts a direct anti-osteosarcoma effect in a dose-dependent manner (Figure 1 and Figure 6). Inhibiting the activity of EGFR tyrosine kinase and consequently retarding its downstream PI3k/Akt and MAPK/ERK pathway (Figure 4; [31]) is likely main molecular mechanism how ZD6474 functions as an effective anti-osteosarcoma drug. These findings will partially account for the reasons that ZD6474 induces G1-S phase arrest and cell apoptosis, in turn, inhibits the proliferation and growth of osteosarcoma cells/xenografts. The inhibitory effects of ZD6474 on osteosarcoma cells *in vitro* are not related with the EGFR levels (Figure 1 and Figure 4A) furtherly suggests that ZD6474 is a multiple-target tyrosine kinase inhibitor and plays anti-pliferation role also via inhibiting other signal pathways.

**Figure 7:**
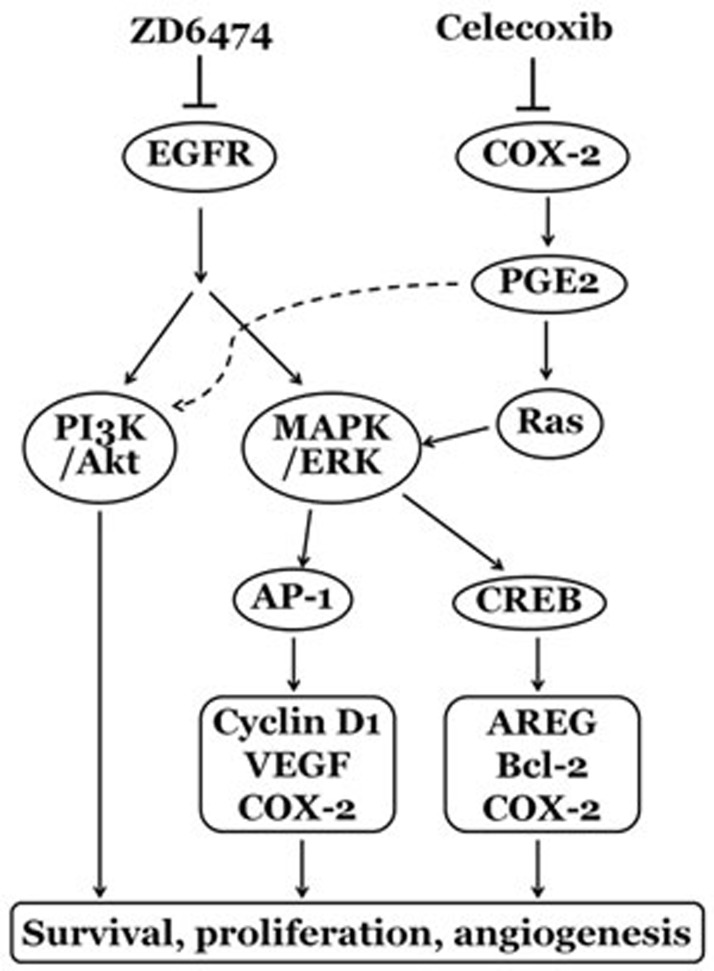
The possible mechanisms of the synergism between ZD6474 and celecoxib on anti- osteosarcoma effects

COX-2 is an enzyme responsible for formation of important biological mediators called prostanoids, including prostaglandins, prostacyclin and thromboxane. Increasing evidence supports the role of COX-2 in promoting tumor cell growth, survival and angiogenesis through the activity of COX-2-derived prostaglandin E2 (PGE2) [35]. PGE2 stimulates prostaglandin E2 receptor to activate MAPK/ERK (also known as the Ras-Raf-MEK-ERK) and PI3K/Akt pathways, and activates multiple transcription factors, such as AP-1 and CREB, which promote the expression of Cyclin D1, VEGF, AREG, Bcl-2, etc., and result in tumor cell proliferation, survival, antiapoptosis and angiogenesis ([36-39]; Figure 7). So COX-2 is considered as a promising therapeutic target for multiple malignancies [35].

High levels of COX-2 expression have been observed in osteosarcoma tissues [20, 21] and was associated with poor prognosis for osteosarcoma patients [22, 23]. Selective COX-2 inhibitors, celecoxib, have been reported to inhibit osteosarcoma growth in previous reports [25, 40] and in our study (Figure 5 and Figure 6). Strangely, U2OS cells with low COX-2 expression display similar sensitivity to celecoxib with other two osteosarcoma cells (MNNG/HOS CL#5 and MG-63) which highly express COX-2 *in vitro* (Figure 5A-5C). It needs more detailed experiments to clarify and explain this phenomenon.

There is now an increasing number of preclinical evidence showing that simultaneous targeting multiple pathways improves the treatment of cancer. In this paper, we also found that the combinative treatment with COX-2 inhibitor celecoxib and EGFR inhibitor ZD6474 presented a synergistic/additive antitumor effect on osteosarcoma *in vivo* and *in vitro* (Figure 5 and Figure 6). Celecoxib was reported to inhibit PI3K/Akt and MAPK/ERK pathways via targeting COX-2 [25, 36, 39], and we found that celecoxib also promoted ZD6474-directed inhibition of MAPK/ERK pathway (Figure 5E). Meanwhile, not only celecoxib but ZD6474 inhibited COX-2 expression via suppressing MAPK/ERK pathway (Figure 5E), which had also been confirmed by the downregulation of COX-2 expression resulted from ERK inhibitor PD98059 (Figure 5F) and upregulation of COX-2 expression induced by epidermal growth factor [41]. These findings may provide a reasonable explanation for the synergism of ZD6474 and celecoxib on anti-osteosarcoma activity.

ZD6474 is a multiple tyrosine kinase inhibitor, so it likely exerted the anti-osteosarcoma effect also through antiangiogenesis or other growth signal pathway [19]. Similarly, celecoxib may play additional antitumor role in osteosarcoma by suppressing angiogenesis and inflammatory response in animal model. These assumptions, supported partially by the report that celecoxib inhibits VEGF expression via suppressing the COX-2/PGE2 pathway [36, 39], need to be confirmed in further study.

In summary, ZD6474 exerts direct antitumor effects on osteosarcoma cells *in vitro* and in xenograft nude mouse model through inhibiting the activation of EGFR pathway and consequently suppressing downstream PI3k/Akt and MAPK/ERK pathways, and the combination of ZD6474 with celecoxib demonstrated a synergistic/additive antitumor effect by the interaction and regulation of EGFR and COX-2/PGE2 pathway (a schematic diagram was shown in Figure 7). These findings shed light on the molecular basis of tyrosine kinase inhibitors alone or in combination with COX-2 inhibitors and a possible new strategy for comprehensive treatment of human osteosarcoma.

## MATERIALS AND METHODS

### Reagents

ZD6474 was synthesized according to a patent (number: wo2,003,039,551) procedure [31-33]. Celecoxib was purchased from Pfizer Inc. (New York, USA). PD98059 was purchased from Sigma (St. Louis, USA). All cell culture materials were purchased from Gibco-Life Technologies (Gaithersburg, MD, USA). Anti-EGFR and COX-2 antibodies were obtained from Signalway Antibody Co., Ltd (Maryland, USA) and Epitomics (Burlingame, USA). Secondary antibodies for Western blot were obtained from Santa Cruz Biotechnology (San Diego, CA). All other antibodies were obtained from Cell Signaling Technology (Danvers, MA, USA). 3-(4,5-dimethylthiazolyl-2)-2,5-diphenyltetrazolium bromide (MTT), RNase, propidium iodide (PI) were purchased from Sigma (St. Louis, USA). Annexin-V/PI binding assay kit was from Invitrogen Ltd. (Paisley, UK), One Step TUNEL Apoptosis Assay Kit was from Keygen BioTECH (Nanjing, China). Other chemicals were from Beyotime Biotechnology Company (Shanghai, China).

### Cell culture

Human osteosarcoma U2OS, MNNG/HOS CL#5, and MG-63 cell lines were from ATCC. These cells were grown in Dulbecco's Modified Eagle's Medium (DMEM) supplemented with 2 mM glutamine, 100 U/ml penicillin, 100 μg/ml streptomycin, and 10% fetal bovine serum (FBS) and cultured in a humidified atmosphere of 5% CO_2_ at 37°C.

### Cell proliferation assay

The cell proliferation was determined by the MTT assay and clone formation assay. For MTT assay, cells were seeded in a 96-well plate at a density of 4000 cells/well, allowed to recover for 24 hours and then treated using complete media alone or containing ZD6474 or celecoxib at different concentrations for 72 hours. Afterwards, 20 μl of MTT solution (5 mg/ml) was added into each well and incubated for 4 hours After removing the medium, 150 μl of DMSO were added per well to dissolve the formazan crystal, and the absorbance was recorded at a 490 nm wavelength using an enzyme-linked immunosorbent assay reader (Molecular Device Inc, Silicon Valley, CA). The data were analyzed using GraphPad Prism 5 software to obtain the IC_50_ (half maximal inhibitory concentration).

For the clone formation assay, cells were seeded in 6-well plates at a density of 500 cells per well, recovered for 24 hours and then treated with ZD6474 at different concentrations. The cells were grown for 10-14 days till there was visible clonal colony formation. The colonies were washed gently with phosphate buffered saline (PBS, 0.01 mol/L, pH 7.4) twice, and fixed and dyed with 5 ml of 0.5% crystal violet solution for 15 min, then washed with PBS and air-dried. Cell proliferation assays were performed at least three times (in replicates of six wells for each data point in each experiment). Monoclone number was counted and data were presented as the mean and standard deviation (SD) for a representative experiment.

### Cell cycle analysis

After ZD6474 treatment for 24 hours, cells were harvested, washed three times with ice-cold PBS, then fixed by resuspension in 75% ice-cold methanol/PBS and incubated overnight at 4°C. After fixing, samples were pelleted at 400 g for 5 min, and the pellets were washed three times with ice-cold PBS, and then resuspended in 500 μl of PBS. After addition of 10 μl of RNase (10 mg/ml), cells were stained with 10 μl of propidium iodide (1 mg/ml) at 37°C for 30 min in the dark. Flow cytometric analysis was performed using a Coulter Epics Elite flow cytometer (Beckman-Coulter, CA, USA).

### Annexin-V/PI binding assay

Floating cells collected by centrifugation and adherent cells harvested by trypsinization were both used for Annexin-V/PI binding assay. After ZD6474 treatment for 48 hours, cells were harvested and washed with ice-cold PBS, resuspended in 100 μl of binding buffer, and stained by adding 5 μl of FITC-Annexin V and 1 μl of PI working solution and incubation at room temperature for 15 min. After diluted with 500 μl of binding buffer, stained cells were immediately analyzed by flow cytometry.

### Western blotting analysis

Total cell lysates were prepared by extracting proteins with RIPA lysis buffer (PBS, 1% NP-40, 0.5% sodium deoxycholate, 0.1% SDS) containing 10 mg/ml aprotinin, leupeptin, and 1 mM phenylmethylsulfonyl fluoride. After incubation on ice for 30 min, samples were centrifuged at 18,000 g for 15 min to remove insoluble materials. Protein concentration in supernatant was measured by Bradford Protein assay. Using 4–12% SDS-polyacrylamide gel, 20-40 μg of total protein was resolved by electrophoresis and was then transferred to a PVDF membrane. The membrane was blocked with either 5% BSA or 5% nonfat milk in Tris-buffered saline followed by incubation with primary antibodies overnight at 4°C with gentle shaking. Then, blots were incubated with horseradish peroxidase-conjugated secondary antibody for 1 hour at room temperature and signals were enhanced by ECL detection system and captured using X-ray film.

### Tumor xenograft models

Female BALB/c nude mice (6-8 weeks old, weight > 18 g) were obtained from the Guangdong experimental animal center (Guangdong, China; license No. SYXK (Yue) 2010-0102). Mice were housed under specific pathogen-free conditions according to protocols approved by the Sun Yat-sen University Institutional Animal Care and Use Committee. After 1-week adaptation, mice were subcutaneously injected in the scapular region with 3×10^6^ MNNG/HOS CL#5 cells in 100 μl of serum-free media. When tumors reached a volume of 100 mm^3^, mice were sacrificed, and the tumors were isolated. Tumors were sheared into small patches (30 mm^3^) and implanted in the scapular region. When tumors reached a volume of 80 mm^3^, the mice with implanted tumors were randomly allocated into groups (*n* = 7 per group) that received (i.g.) ZD6474 (50, 100, or 200 mg/kg/day), celecoxib (150 mg/kg/day), ZD6474 (100 mg/kg/day) and celecoxib (150 mg/kg/day), or vehicle (sterilized water) once daily at a dose of 0.1 ml/10 g body weight. Tumor volume was assessed every three days by caliper measurement of tumor diameter and calculated according to the formula V = L ×W^2^/2 (L, length; W, width). After 22 days of treatment, mice were sacrificed, and tumors were resected and weighed. All animal experiments were conducted in accordance with the “Guidelines for the Welfare of Animals in Experimental Neoplasia”.

### TUNEL assays

The tumor tissues stripped from xenograft nude mice were fixed with 10% formaldehyde, followed by paraffin embedding and section, and then terminal deoxyribonucleotidyl-transferase-mediated dUTP nick-end labeling (TUNEL) assays were performed according to the manufacturer's instructions for the assessment of cell apoptosis in the sections. Labeled cells were then counted in 10 random fields of 200× magnification per study group.

### Statistical analysis

The data were evaluated using ANOVA with SPSS16.0 software. The combined effect of the drugs was assessed with the Q value using Zheng-Jun Jin's method as previously described [42-44]: Q = E_AB_/[E_A_+E_B_(1-E_A_)] (E_A_, E_B_ and E_AB_ indicate the inhibitory rates of A, B and the combination of the two drugs). The effect of the combination of two drugs can be classified as antagonistic (Q < 0.85), additive (0.85 < Q < 1.15), or synergistic (Q>1.15).
